# Comparative Study of the Dehydrothermal Crosslinking of Electrospun Collagen Nanofibers: The Effects of Vacuum Conditions and Subsequent Chemical Crosslinking

**DOI:** 10.3390/polym16172453

**Published:** 2024-08-29

**Authors:** Ján Kužma, Tomáš Suchý, Lukáš Horný, Monika Šupová, Zbyněk Sucharda

**Affiliations:** 1 Faculty of Mechanical Engineering, Czech Technical University in Prague, Technická 4, Prague 6, 160 00 Prague, Czech Republic; suchy@irsm.cas.cz (T.S.); lukas.horny@fs.cvut.cz (L.H.); 2 Institute of Rock Structure and Mechanics of The Czech Academy of Sciences, v. v. i., V Holešovičkách 94/41, Prague 8, 182 09 Prague, Czech Republic; supova@irsm.cas.cz (M.Š.); sucharda@irsm.cas.cz (Z.S.)

**Keywords:** Collagen, crosslinking, dehydrothermal crosslinking, chemical crosslinking, EDC/NHS, uniaxial tensile tests, swelling, degradation

## Abstract

Collagen nanofibrous materials have become integral to tissue engineering due to their exceptional properties and biocompatibility. Dehydrothermal crosslinking (DHT) enhances stability and maintains structural integrity without the formation of toxic residues. The study involved the crosslinking of electrospun collagen, applying DHT with access to air and under vacuum conditions. Various DHT exposure times of up to 72 h were applied to examine the time dependance of the DHT process. The DHT crosslinked collagen was subsequently chemically crosslinked using carbodiimides. The material crosslinked in this way evinced elevated Young’s modulus values and ultimate tensile strength values, a lower swelling rate and lower shrinkage ratio during crosslinking, and a higher degree of resistance to degradation than the material crosslinked solely with DHT or carbodiimides. It was shown that the crosslinking mechanism using DHT occupies different binding sites than those using chemical crosslinking. Access to air for 12 h or less did not exert a significant impact on the material properties compared to DHT under vacuum conditions. However, concerning longer exposure times, it was determined that access to air results in the deterioration of the properties of the material and that reactions take place that occupy the free bonding sites, which subsequently reduces the effectiveness of chemical crosslinking using carbodiimides.

## 1. Introduction

Collagen nanofibrous materials have emerged as a pivotal component in the field of tissue engineering owing to their remarkable properties and biocompatibility [1,2,3,4,5,6,7,8]. The history of collagen nanofibrous materials traces back to the early 1990s when researchers began exploring electrospinning techniques aimed at producing nanofibrous scaffolds. Electrospinning enabled the fabrication of collagen nanofibers with high surface area-to-volume ratios that resemble the natural extracellular matrix (ECM) of tissues [9,10,11,12,13]. Over time, advancements in nanotechnology and the biomaterial sciences have led to the improvement of the fabrication processes and properties of collagen nanofibrous materials. These materials are used in a wide variety of tissue engineering applications including wound healing, bone regeneration, cartilage regeneration, skin tissue regeneration and cardiovascular repair [2,3,5,14,15]. Collagen suspensions can be easily prepared and used as delivery vectors for macro and micro drug molecules [16]. Via the mimicking of the native ECM, recent advances in the manufacture of collagen nanofibrous scaffolds have enhanced both structural support and cues for cell adhesion, proliferation and differentiation, thus facilitating tissue regeneration and repair with promising clinical implications [7,11,13,17].

The structure of collagen is significantly disturbed following the electrospinning process as manifested, for example, by a decrease in stability in the aqueous environment or impaired mechanical properties, which greatly limit its application. Therefore, it is necessary to crosslink this material in order to restore its properties [18,19,20,21,22]. Several chemical and physical collagen crosslinking methods are available that entail the formation of covalent bonds between the collagen molecules so as to bolster both the stability of the material and its mechanical properties [20,23]. Chemical crosslinkers include glutaraldehyde and formaldehyde; moreover, a new trend has emerged toward the adoption of natural agents in the crosslinking of electrospun collagen based materials such as N-(3-dimethylaminopropyl)-N-ethylcarbodiimide hydrochloride (EDC)/N-hydroxysuccinimide (NHS) and genipin [24,25]. Chemical agents react with the amino acid residues in the collagen to establish stable bonds, while physical methods such as dehydrothermal crosslinking (DHT), UV and gamma irradiation induce crosslinking via energy-mediated processes [20,23,25,26,27,28,29]. The application of these techniques leads to the enhanced tensile strength and stiffness of collagen materials and elevated resistance to enzymatic degradation [24].

DHT presents numerous benefits with respect to collagen-based materials. It ensures the elimination of water from collagen scaffolds at elevated temperatures and facilitates intermolecular interactions and crosslinking between the collagen molecules. This procedure enhances both the stability of the material and its mechanical characteristics while preserving its morphology, biocompatibility and bioactivity [30]. DHT provides a solvent-free and gentle crosslinking approach, minimizes the potential for the formation of toxic residues and maintains the inherent structure of the collagen [18,19,31]. Nevertheless, despite its favorable attributes, the DHT crosslinking process is not yet fully understood. 

The various types of crosslinking processes employed for collagen-based materials operate via the formation of covalent bonds between the collagen molecules [23,28,30]. However, it remains unclear whether these processes share identical binding mechanisms at the molecular level. Consequently, questions arise regarding the occupation of the same binding sites within the collagen molecule during physical and chemical crosslinking. Moreover, the role of the access of air during the DHT crosslinking process and its impact on the availability of free binding sites crucial for the crosslinking process requires clarification. A comprehensive understanding of the mechanism underlying DHT crosslinking holds significant promise in terms of advancing the development of nanostructured collagen materials. Such an understanding would enable the precise tailoring of the properties of materials so as to better align with specific practical applications, thus enhancing the efficacy and versatility of collagen-based constructs in the tissue engineering and regenerative medicine domains. 

The shrinkage of nanostructured collagen layers during crosslinking is an important parameter that needs to be considered with concern to application. Shrinkage may result in the cracking of the surface layer of implants or cause internal stress within the scaffold, which potentially leads to the failure thereof. The careful choice of the crosslinking conditions with respect to the purpose of the layer has the potential to contribute significantly to the compactness and integrity of the resulting coatings and structures.

The aim of this study was to determine the impact of the crosslinking of electrospun collagen via DHT with access to air or under vacuum conditions. The crosslinking efficiency and the type of newly formed bonds were subsequently evaluated via chemical crosslinking using EDC/NHS. This experimental approach aimed to verify whether amide crosslinks or covalent bonds are formed within the collagen during DHT crosslinking. The experiment also aimed to verify whether the application of EDC/NHS to DHT crosslinked layers is still able to lead to the formation of new crosslinks and how this process is affected by access to air. Aimed at determining the effectiveness of the methods applied, collagen layers were prepared via electrospinning using the procedure verified in our previous studies [32,33,34,35]. Electrospun collagen was crosslinked using DHT with access to air or under vacuum conditions for up to 72 h. The physically crosslinked layers were further chemically crosslinked using EDC/NHS for up to 72 h. The extent of crosslinking and the impact of each of the crosslinking parameters were evaluated by means of uniaxial tensile testing, the determination of the extent of degradation, the swelling ratio in aqueous media, the material shrinkage ratio, scanning electron microscopy and infrared spectroscopy.

## 2. Materials and Methods

### 2.1. Preparation of the Electrospinning Solution

Collagen nanofibrous materials were prepared based on an 8 wt% collagen (VUP Medical, Brno, Czech Republic) solution. The collagen was dispersed in phosphate buffer saline (PBS, Sigma-Aldrich, Saint Louis, MO, USA), to which polyethylene oxide was added as an auxiliary polymer (PEO, Mn 900,000, Sigma-Aldrich, Taufkirchen, Germany) in the amount of 8 wt% to the weight of the collagen. The solution was placed in an incubator for 48 h at 37 °C, whereupon the solution was homogenized and ethanol was added at a ratio of 1:1 to the weight of the PBS [32,33].

### 2.2. Electrospinning

The electrospun materials were fabricated using a high voltage of 45 kV with a feeding rate of 80 μL·min^−1^. The temperature was maintained at (24 ± 3) °C, the distance between the needle and the collector was set at 20 cm and the relative humidity was maintained at 23–26% (4SPIN, Contipro, Dolní Dobrouč, Czech Republic). The electrospinning process was enhanced via its combination with electro blowing. The flow rate of the preheated air (25 °C) was set at 10 L per minute. All the electrospun materials were collected on a rotary collector in the form of a cylinder with a rotation speed of 310 rpm; the resulting dimensions of the spun layer were 22 cm × 36 cm [33,34].

### 2.3. Crosslinking

The stability of all the collagen layers was modified by means of dehydrothermal and/or chemical crosslinking. The DHT crosslinking was divided into two groups, the first with the supply of atmospheric air and the second under vacuum conditions. Crosslinking took place at 150 °C and lasted for 3, 6, 12, 24, 48 and 72 h for both groups. Subsequent chemical crosslinking was performed in 200 mL of 95% ethanol solution per gram of collagen [3]. The solution containing EDC had a molar ratio of 5:8 to the collagen and those containing EDC and NHS a molar ratio of 4:1. The same crosslinking solution was used for all the chemically crosslinked samples. EDC and NHS (Sigma Aldrich, Taufkirchen, Germany) were used as received. Following the reaction period, all the layers were washed in 0.1 M Na_2_HPO_4_ (2 × 45 min) and subsequently rinsed with deionized water (30 min). The layers were then frozen at −15 °C for 5 h and lyophilized (BenchTop 4KZL, VirTis, Los Angeles, CA, USA).

Three sets of control groups were used for comparison purposes: the source of the collagen for the preparation of all the samples, i.e., collagen lyophilizate (OR), electrospun non-crosslinked collagen (ES) and electrospun collagen crosslinked with EDC/NHS (see Table 1). The control samples were selected in order to evaluate the effect of the dissolution of the collagen (OR) and the effect of electrospinning (ES) and for comparison with the EDC/NHS crosslinking method; this method is widely applied and was verified in several of our previous studies as suitable for application under both in vitro and in vivo conditions [32,33,34,35]. The selection of the control samples was deliberate and aimed at discerning the impact of the DHT method on the material with the maximum degree of efficacy. 

### 2.4. Mechanical Testing

Aimed at verifying the impacts of the differing crosslinking approaches on the mechanical properties of the collagen electrospun material, rectangular specimens with typical dimensions of 40 mm × 10 mm × 1 mm (length × width × thickness) were subjected to uniaxial tensile testing. The experiments were conducted using a Zwick/Roell multipurpose testing machine with a built-in video extensometer. The video extensometer automatically ascertained the reference length and elongation of the investigated parts of specimens during experimentation. The tensile tests were conducted at a constant clamp velocity of 0.1 mm·s^−1^; the loading force was measured using a U9C (±25 N, HBM, Darmstadt, Germany) force transducer. The deformation, *ε*, of the specimens was expressed by means of Equation (1), where *L* denotes the deformed length determined by the video-extensometer, and *L*_0_ is the reference length.

(1) $$\varepsilon =\frac{L-L_{0}}{L_{0}}$$

(2) $$\sigma =\frac{F}{A_{0}}$$

The mechanical stress, *σ*, that the specimens were able to bear during loading is expressed in Equation (2). The so-called nominal stress tensor was used in our analysis, and the tensile component thereof is given as the ratio of the acting force, *F*, to the reference cross-section area, *A*_0_. The tensile test procedure was performed by means of five preloading cycles so as to attain the preconditioned (repeatable) mechanical response. The sixth loading cycle was conducted up to failure; its preconditioned component was analyzed during the subsequent mathematical modeling phase. The ultimate tensile strength (UTS) was calculated as the maximum *σ* attained in the experiment. The experiments were conducted in air and at room temperature with the specimens in the hydrated state (1 h of immersion in the physiological solution prior to measurement).

### 2.5. Model for the Stress–Strain Relationship

A hyperelastic material model was employed to describe the nonlinear mechanical response of the hydrated collagen layers. The particular form of the strain energy density function *W* that was applied is described in Equation (3), i.e., the Fung–Demiray exponential model [16], which is commonly used in the field of soft tissue biomechanics.

(3) $$W=\frac{\mu }{2\alpha }\left(e^{\alpha \left(\lambda_{1}^{2}+\lambda_{2}^{2}+\lambda_{3}^{2}-3\right)}-1\right)$$

*μ* and *α* are the material parameters in Equation (3). The kinematics of the uniaxial tensile test were assumed as described by Equation (4), where ***X*** = (*X*_1_, *X*_2_, *X*_3_)*^T^* is the position vector of a particle of the material in the reference configuration and ***x*** = (*x*_1_, *x*_2_, *x*_3_)*^T^* is the position vector of the same particle in the deformed state. In this case, the deformation gradient **F**, which is defined as **F** = *∂**x**/∂**X***, has the form **F** = diag[*λ*_1_, *λ*_2_, *λ*_3_], where *λi* (*i* = 1, 2, 3) are the so-called principal stretches.

(4) $$x_{1}=\lambda_{1}X_{1}\;x_{2}=\lambda_{2}X_{2}\;x_{3}=\lambda_{3}X_{3}$$

Concerning hyperelastic materials, the strain energy density function (3) serves as a potential function for the stress. The stress tensor is obtained as a derivative of *W* with respect to the strain tensor. In the case of the nominal (also referred to as the first Piola-Kirchhoff) stress tensor ***σ***, this relationship is expressed via Equation (5).

(5) $$\sigma =\frac{\partial W}{\partial F}-pF^{-1}$$

Concerning Equation (5), the assumption of the incompressible behavior of collagenous materials was adopted, i.e., that *p* in Equation (5) is an undetermined multiplier that enforces incompressibility. The final Equation for the mechanical stress carried by the material under uniaxial tension was obtained (6) by combining (3), (4), (5) and the incompressibility condition *det*(**F**) = 1. The first Piola–Kirchhoff in (6) is expressed as the function of *ε*, which is obtained from the principal stretch in the direction test *λ* via *λ* = *ε* + 1.

(6) $$\sigma =\mu \left(1+\varepsilon -\frac{1}{{\left(1+\varepsilon \right)}^{2}}\right)e^{\alpha \left({\left(1+\varepsilon \right)}^{2}+\frac{2}{1+\varepsilon }-3\right)}$$

The parameters *α* and *μ* were estimated applying the least squares method in which the deviations between the experimentally measured stress (2) and the stress expressed by the theoretical model (6) were minimized. In addition to the model parameters, the (initial) Young’s modulus values were determined as derivatives of the model stress-strain curve at the origin (at *ε* = 0).

### 2.6. Degradation and Swelling

The degree of crosslinking was also indirectly assessed by determining the extent of degradation and swelling, for the purpose of which samples (*n* = 10) were immersed in deionized water at 37 °C for 24 h. The degradation of the collagen layers was evaluated by means of the determination of the mass loss (*D*), which was calculated according to Equation (7) where *W*_0_ is the initial dried weight of the sample and *W_t_* is the dried weight of the sample following immersion. Drying following immersion was performed via the lyophilization of the frozen samples (−15 °C).

(7) $$D=\frac{W_{t}}{W_{0}}\times 100\%$$

The swelling ratio (*E_s_*) was calculated applying Equation (8), where *W_sw_* is the weight of the swollen sample. The weight of the swollen samples was measured following the removal of each sample from the medium and after a 1 min delay and the removal of any excessive medium surrounding the sample.

(8) $$E_{s}=\frac{W_{sw}-W_{0}}{W_{0}}$$

### 2.7. Material Shrinkage

The layers were cut into 5 × 5 cm squares and placed between two glass surfaces and crosslinked by means of DHT and DHT under vacuum conditions, and subsequently using EDC/NHS. The shrinkage ratio (*S*) of the samples was measured using a high-resolution camera in the initial state *A*_0_, following DHT for 72 h, and after EDC/NDS crosslinking in the wet state *A_W_*, and calculated applying Equation (9). The images were evaluated by means of image analysis (NIS Elements imaging software version 4.13 and the ImageJ version 1.8.0 program https://imagej.net/ij/).

(9) $$S=\frac{{A_{0}-A}_{w}}{A_{0}}\times 100\%$$

### 2.8. SEM Image Analysis

The inner structures of the specimens were characterized by means of scanning electron microscopy (STEM Apreo S2 microscope, Thermo Scientific, Waltham, MA, USA)) in the high vacuum mode on ETD. The specimens were cut using a surgical knife prior to the SEM analysis. The resulting cross-sections were mounted on stubs using carbon adhesive stickers and sputter coated with Pt in an Ar atmosphere. (Leica EM ACE600, Specion sro., Praha, Czech Republic). Ten micrographs were taken randomly (mag. 10,000×) for the qualitative evaluation of the morphology.

### 2.9. Infrared Spectroscopy

The secondary structure collagen materials were evaluated by means of attenuated total reflection infrared spectrometry (ATR-FTIR) using an iS50 infrared spectrometer (Nicolet Instrument, Madison, WI, USA) equipped with an ATR device with a diamond crystal. All the materials were measured in the lyophilized state. All the spectra were recorded in absorption mode in the range 4000–400 cm^−1^ at a resolution of 4 cm^−1^ and 64 scans. The samples were measured 10 times so as to verify the homogeneity of the material. The spectra were processed by means of OMNIC version 9 software (Thermo Scientific, Waltham, MA, USA). The areas of the amide I bands were deconvoluted; the number of bands and their positions were predetermined by applying a combination of the Fourier self-deconvolution procedure and the secondary derivative method. The areas of the amide I bands and the various area/intensity ratios were subsequently statistically evaluated.

### 2.10. Statistical Evaluation

The statistical analysis was performed in GraphPad Prism software (ver. 9.5.0 (730), GraphPad Software, San Diego, CA, USA). The normality of the data was verified applying the Shapiro–Wilk test and the construction of Q–Q plots. The homoscedasticity was verified applying the Bartlett and Brown–Forsythe tests. Non-parametric analysis was employed since the assumption of normality or homoscedasticity was violated. The Kruskal–Wallis test was performed with a subsequent post hoc test based on the Dunn test (with or without correction for multiple comparison, depending on the method of comparison). The Mann–Whitney two-tailed test was employed for the two-sample comparison. Statistical significance was accepted at *p* ≤ 0.05. Scatter plots with the median and interquartile range (IQR) were employed for the graphical presentation of the data.

## 3. Results

### 3.1. Mechanical Testing

A total of 250 uniaxial tensile tests was carried out, some of which, however, failed prior to the completion of the testing procedure; this was the case of the 3 h DHTvac group, concerning which none of the 10 tests yielded a successful outcome. Hence, this group was not included in the subsequent evaluation.

**Figure 1:**
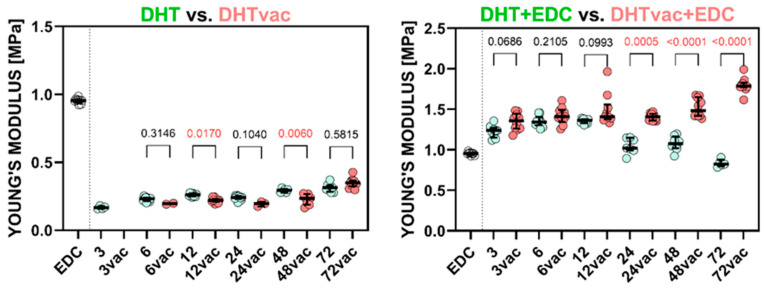
Comparison of the Young’s modulus of the collagen layers treated via DHT and DHTvac, and DHT+EDC and DHTvac+EDC. Statistically significant differences are indicated by *p*-values ≤ 0.05 (highlighted in red; Dunn’s test without correction, *n* = 2–10). EDC was used as the control group. The results are presented in the form of scatterplots in which each point represents one observation; the line represents the median and the whiskers the IQR.

**Figure 2:**
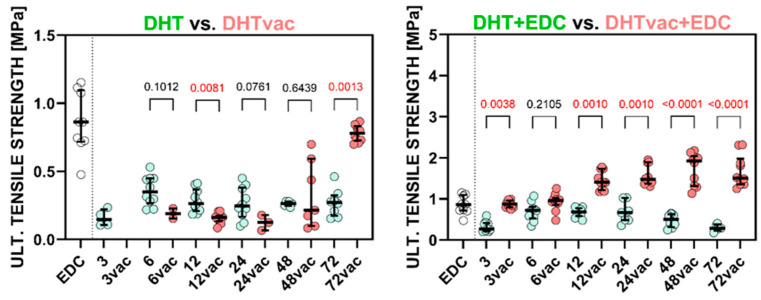
The ultimate tensile strength of the collagen layers; comparison of DHT versus DHTvac and DHT+EDC versus DHTvac+EDC. Statistically significant differences are indicated by *p*-values ≤ 0.05 (highlighted in red; Dunn’s test without correction, *n* = 2–10). EDC was used as the control group. The results are presented in the form of scatterplots in which each point represents one observation; the line represents the median and the whiskers the IQR.

The scatter plots in Figure 1 depict the stiffness determined for the DHT versus DHTvac groups and the DHT+EDC and DHTvac+EDC groups. The EDC/NHS (referred to as EDC) results are also included for the sake of comparison with the standard crosslinking method. The significant statistical differences used for the *p*-values of the Dunn’s tests are indicated in red. Figure 2 illustrates the ultimate tensile strength. The numerical values of the Young’s modulus and UTS are presented in Table 2. 

The DHT exhibited greater compliance and lower strength than the EDC/NHS; the same applied for the DHTvac with the exception of the 72 h of crosslinking treatment. Concerning the stiffness, the DHT+EDC evinced a higher Young’s modulus than the EDC/NHS; however, it also evinced a lower UTS. Nevertheless, higher degrees of stiffness and ultimate tensile strength than observed for the EDC/NHS control were exhibited by the layers treated with DHTvac+EDC (Young’s modulus of (1.921 ± 0.093) MPa after 72 h of the crosslinking procedure, and UTS of (2.736 ± 0.748) MPa after 48 h).

Regarding the evaluation of the access of air during the DHT crosslinking process, the Young’s modulus values of the DHT and DHTvac differed only slightly, and no statistical significance was recorded in many cases, in a similar way to the UTS. In contrast, with respect to the comparison of the DHT+EDC where the DHT was conducted in the presence of air, and DHTvac+EDC where the DHT was conducted under vacuum conditions, both Figure 1 and Figure 2 indicate a change in the response behavior for treatment times in excess of 12 h, following which, the DHTvac+EDC was, in all cases, significantly stiffer and stronger.

### 3.2. Model

**Figure 3:**
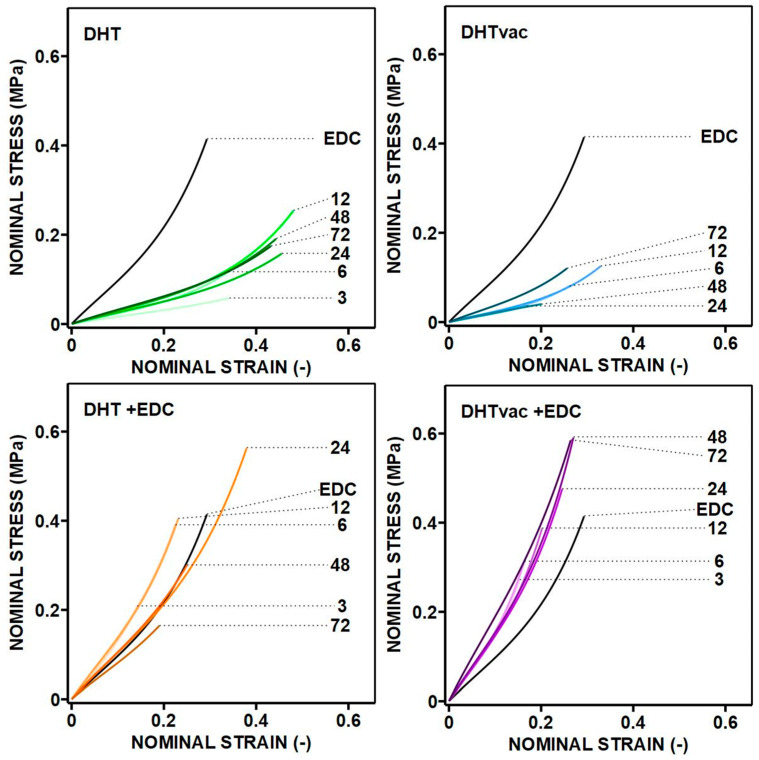
Comparison of the models for the various groups: DHT, DHTvac, DHT+EDC, DHTvac+EDC and the EDC/NHS control (shown as EDC in the graphs). The numbers shown in the figures indicate the crosslinking time.

Figure 3 depicts the stress–strain relationships determined by the uniaxial tensile testing of the hydrated collagen materials. Their mechanical response is non-linear and the Fung–Demiray model (3) fitted well to the data in all cases. As shown in Table 2, the values of the coefficient of determination *R*^2^ were no lower than 0.966 for any of the groups. The estimated values of the model parameters *α* and *m* are presented in Table 2 with the confidence intervals (95% level) for the regression model.

The stress–strain curves indicated that the application of DHT alone was in all cases more response compliant than the EDC/NHS; the same applied to DHTvac. The DHT+EDC evinced stress–strain curves that, in some cases, lay above (were stiffer than) the EDC/NHS, but in other cases, the DHT+EDC curves lay below (were stiffer than) the mechanical response of the control group. The DHT without access to air that was subsequently crosslinked with EDC/NHS (denoted DHTvac+EDC) evinced a mechanical response that was in all cases stiffer than the mechanical response of the electrospun collagen crosslinked by EDC/NHS alone.

### 3.3. Degradation Test

**Figure 4:**
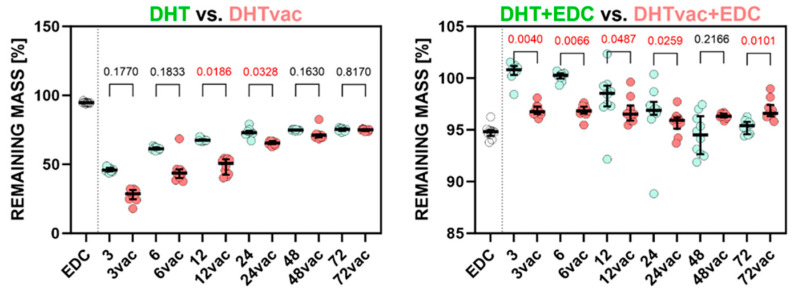
The remaining mass following the degradation testing of the collagen materials compared to the samples treated via the DHT versus DHTvac and the DHT+EDC versus DHTvac+EDC processes. Statistically significant differences are indicated by *p*-values ≤ 0.05 (highlighted in red; Dunn’s test without correction, *n* = 8–10). The results are presented in the form of scatterplots in which each point represents one observation; the line represents the median and the whiskers the IQR.

The results presented in Figure 4 underscore the importance of crosslinking following collagen electrospinning. The non-crosslinked electrospun materials in the aqueous milieu underwent complete degradation within 24 h and could not be directly compared to the crosslinked materials. Chemical crosslinking using EDC/NHS (standard), which served as the control, evinced a remaining mass of 94.78% and enhanced material properties and stability compared to the electrospun non-crosslinked material (which was completely degraded), as substantiated via in vitro and in vivo testing [13,36].

All the physical and chemical collagen crosslinking methods bolstered resistance to degradation [6]. The degree of crosslinking can, thus, be regarded as indicative of the resilience of collagen to degradation in aqueous environments, as manifested by the reduced mass loss. As illustrated in Figure 4, the electrospun layer without crosslinking evinced substantial dissolution and complete degradation, whereas the materials subjected to DHT and DHTvac demonstrated elevated resistance proportionate to the duration of exposure. The DHTvac appears to be marginally less effective, as indicated by the higher weight loss, which stabilized after approximately 24 h at a mass loss of 65.42%. Notably, subsequent chemical crosslinking proved to be more efficacious for the DHT in air, as indicated by the higher weight loss evinced by the DHTvac samples up to 48 h.

Both the DHT and DHTvac groups crosslinked with EDC/NHS exhibited almost complete resistance to degradation with the retention of approximately 96% of the mass, which was similar to the control sample crosslinked with EDC/NHS only. Figure 4 illustrates the statistically significant differences, albeit with a minimal practical impact.

### 3.4. Swelling Test

The elevated density of crosslinks results in a more densely packed structure with diminished swelling capacity. Electrospun collagen subjected to higher degrees of crosslinking tends to exhibit higher resistance to swelling than its less crosslinked or non-crosslinked counterparts [37].

**Figure 5:**
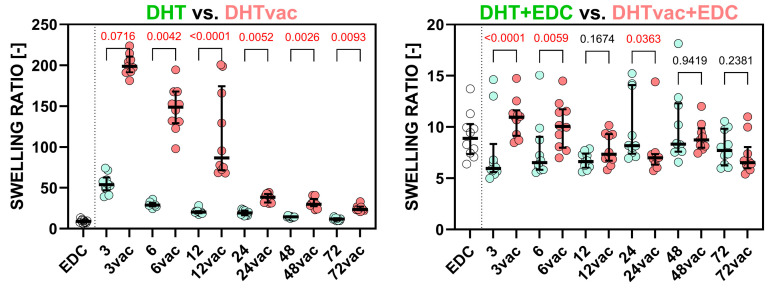
Swelling ratio of the collagens compared with the samples treated via the DHT versus DHTvac and DHT+EDC versus DHTvac+EDC processes. Statistically significant differences are indicated by *p*-values ≤ 0.05 (highlighted in red; Dunn’s test without correction, *n* = 8–10). The results are presented in the form of scatterplots in which each point represents one observation; the line represents the median and the whiskers the IQR.

As depicted in Figure 5, swelling diminished with increasing exposure time for the DHT method with respect to both the vacuum and air conditions, thus indicating an escalating degree of crosslinking. The swelling ratios began to stabilize after DHT treatment exceeding 6 h (18.18 times its mass) and DHTvac treatment exceeding 24 h (24.81) 

The DHT and DHTvac methods demonstrated similar swelling degrees when crosslinked in conjunction with EDC/NHS, in a similar way to the control sample crosslinked solely with EDC/NHS (8.66). This highlighted chemical crosslinking as a highly effective strategy for reducing swelling rates, whether employed individually or in combination with DHT or DHTvac.

Figure 5 shows that the DHT process conducted in the presence of air is more efficient than that conducted under vacuum conditions. Comparable levels of crosslinking were attained after 24 h of DHT treatment under vacuum conditions compared to treatment in air. Concerning the combination of DHT and DHTvac with EDC/NHS, minor statistically significant differences were observed; however, their actual impact was less than 20%, which was negligible compared to the comparison between DHT and DHTvac.

### 3.5. Shrinkage of the Material

**Figure 6:**
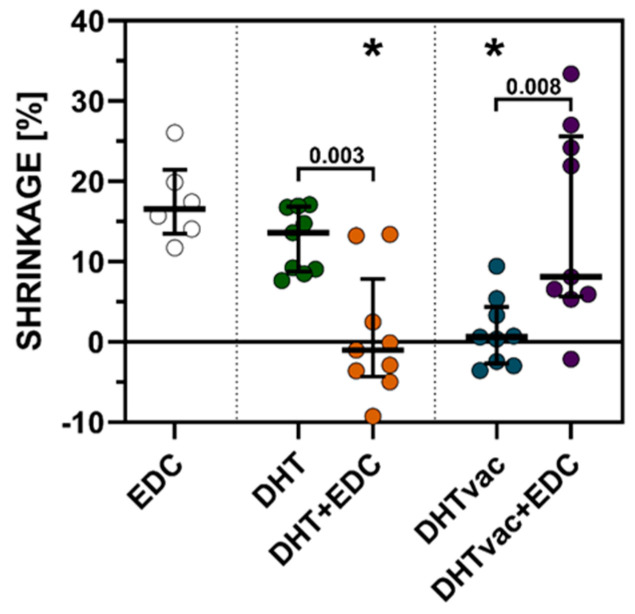
Shrinkage ratios of the collagen layers crosslinked with EDC/NHS, DHT in air, DHT under vacuum conditions and a combination of DHT in air and under vacuum conditions with subsequent EDC/NHS crosslinking. * indicates the comparison with EDC (corrected Dunn’s test); the *p*-values in the graph represent the comparison of the individual DHT methods in combination with EDC/NHS crosslinking (Mann–Whitney test), *n* = 6–9. The results are presented in the form of scatterplots in which each point represents one observation; the line represents the median and the whiskers the IQR.

The control samples crosslinked chemically via EDC/NHS exhibited an approx. 16% reduction in their area (see Figure 6). The samples exposed to air were comparable to the control. The reduction was particularly evident when compared to the vacuum samples, which exhibited almost no significant shrinkage. Following subsequent chemical crosslinking with EDC/NHS, the DHT crosslinked samples with access to air experienced an increase in the surface area to approach the original area, while the vacuum DHT crosslinked samples exhibited shrinkage of around 8%.

### 3.6. SEM Morphology Analysis

**Figure 7:**
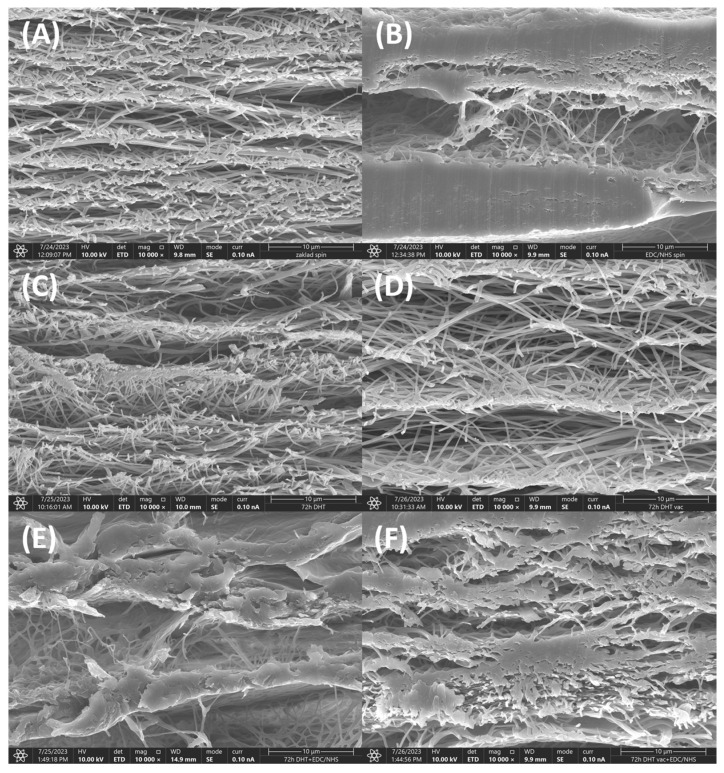
Images of the locations at which the collagen structure has degraded and a film has partially formed compared to the non-chemically crosslinked samples (**A**) Control sample, non-crosslinked electrospun layer; (**B**) Control sample crosslinked with EDC/NHS; (**C**) DHT, exposure time of 72 h; (**D**) DHTvac, exposure time 72 h; (**E**) DHT, exposure time 72 h with EDC/NHS; (**F**) DHTvac, exposure time 72 h with EDC/NHS.

An example of the SEM analysis of the collagen layers is shown in Figure 7. Figure 7A illustrates an electrospun collagen layer prior to crosslinking. The fibrous structure is clearly visible without any hints of the presence of foil. The other panels (Figure 7B–E) also serve to prove that the fibrous structure was preserved within the EDC, DHT 72 h, DHTvac 72 h and DHT+EDC 72 h treatment. In contrast, Figure 7F illustrates the DHTvac+EDC after 72 h of treatment in which the partial fusing of the fibers is evident.

### 3.7. Infrared Spectrometry

FTIR enables the interpretation of changes in the structure of collagen following electron irradiation. The secondary protein structure embodies five amidic bands in the FTIR spectra [38]. Amide A associated with NH_2_ stretching is visible at 3300 cm^−1^; however, this band also contains hydrogen bonds from intermolecular water. Amide B at ∼3070 cm^−1^ is ascribed to the stretching vibrations of the N-H bonds in the secondary amides, as well as to C-H stretching in the sp_2_ hybridization. The Amide I band originated from C=O stretching vibrations coupled with N–H bending vibrations and the amide II bands originated from NH_2_ bending vibrations coupled with C–N stretching vibrations. Amide III (at ∼1205, 1240 and 1280 cm^−1^) together with the band at 1340 cm^−1^ provides further proof of the existence of a triple helical structure in the collagen [39,40]. The comparison of the infrared spectra of the collagen materials exposed to all the crosslinking processes performed in both environments (air and vacuum) for 72 h prior to swelling and their comparison with the original, electrospun and polyethyleneoxide (PEO) are shown in Figure S1 in the Supplementary File. 

As can be seen from Figure S1, the spectra of the collagen materials evince visible changes corresponding to the presence of PEO (the red arrows), with the exception of the samples that were crosslinked with EDC/NHS, concerning which, the PEO was washed out during the crosslinking process.

The first change related to the slight increase in band ∼3500 cm^−1^ was ascribed to the O-H bonds. The wide band from 3150 to 3650 cm^−1^ represents a mutual band of amide A and several OH group modes: free OH groups and the intramolecular and intermolecular H-bridges of the OH groups [41,42]. A further wide band at ∼1720 cm^−1^ relates to the C=O bonds in the ketones and carboxyls. These changes resulted from the application of a temperature of 150 °C, and they were more apparent in the case of the application of air environment. The spectral region at 2800–3000 cm^−1^ related to the C-H aliphatic bonds, and the triplet peak in the 1000 to 1200 cm^−1^ region related to the C-C and C-O stretching vibrations in the PEO [43].

Changes in the secondary (the hydrogen bond patterns between the main-chain peptide groups as described according to two main types, i.e., the α-helix and the β-sheets) structure of collagen may occur during the crosslinking process. These changes are best assigned via the deconvolution of the amide I band. The application of this procedure for the spectra of all the studied materials revealed four bands, i.e., at ∼1615, 1630–1635, 1660 and 1685–1690 cm^−1^, which differed in terms of their intensity. All the resulting bands were expressed as percentages of the total amide I area. The band at ∼1660 cm^−1^, which related to the triple helix, comprises the main principle spectral feature for the secondary structure of collagen, while the other bands (at ∼1630–1635 cm^−1^, at ∼1610–1615 cm^−1^ and at ∼1690 cm^−1^) represent other structures present in collagen, i.e., the left-handed 3–10 helix in the denaturated state, the spectral manifestation of the aromatic amino acids in the disintegrated collagen state, e.g., gelatin [44] and the β-turn and the antiparallel β-sheet structure [45], respectively.

A statistically significant decrease in the 1660 area occurred with respect to all the materials compared to the original (OR) collagen (Figure S2 in the Supplementary File). The collagens that were also chemically crosslinked (DHT+EDC and DHTvac+EDC) also evinced a statistically significant decrease (of below 40%) in this area compared to the electrospun (ES) collagen. It is evident that EDC contributes to the disruption of the triple helices and, thus, contributes to the changes in the other structural states.

**Figure 8:**
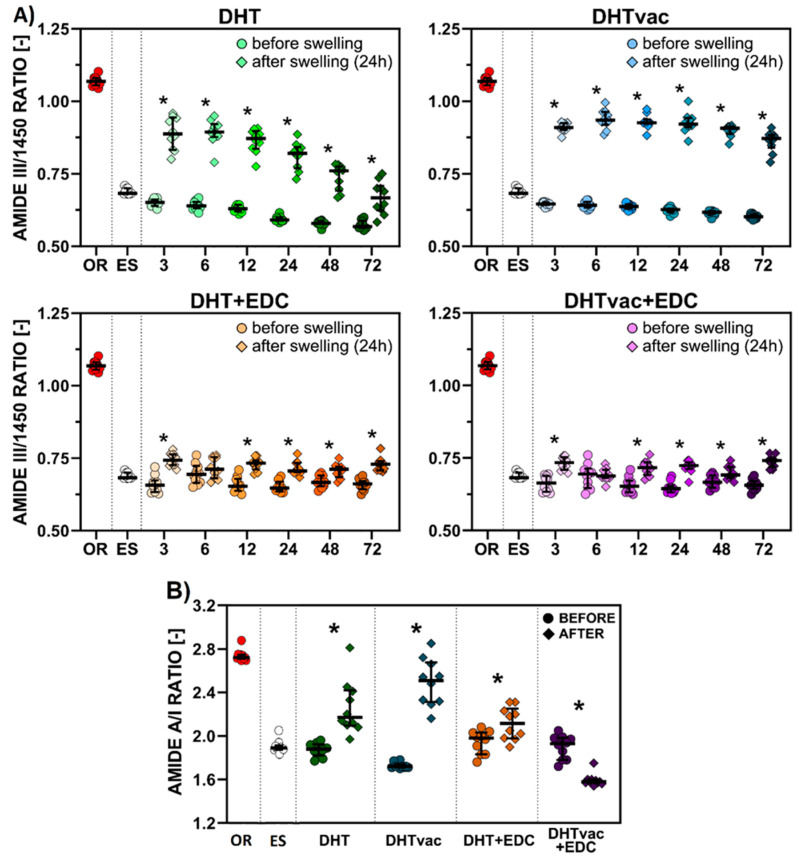
(**A**) Amide III/1450 ratios of the original (OR) and electrospun (ES) collagens compared with the samples treated via the DHT, DHTvac, DHT+EDC and DHTvac+EDC processes. The pairs with *p*-values ≤ 0.05 (Mann–Whitney two-tailed nonparametric test, *n* = 10) are marked “*” for the comparison of the samples before and after exposure in distilled water for 24 h; (**B**) Amide A/I ratio of the original (OR), electrospun (ES) collagens and the collagen samples treated via the DHT, DHTvac, DHT+EDC and DHTvac+EDC processes after 72 h. The pairs with *p*-value ≤ 0.05 (Mann–Whitney two-tailed test, *n* = 10) are marked “*” for the comparison of the samples before and after exposure in distilled water for 24 h. The results are presented in the form of scatterplots in which each point represents one observation; the line represents the median and the whiskers the IQR.

Amide III is very sensitive to the presence of tertiary (the 3D structure created by a single protein polypeptide chain that may include one or several domains) and quaternary (the 3D structure composed of the aggregation of at least two individual polypeptide chains that operate as a single functional unit) structures of native collagen. The ratios of the peak intensity of amide III (∼1240 cm^−1^) to 1450 cm^−1^ (assigned to the pyrrolidine ring vibrations of proline and hydroxyproline) can be considered as markers of the integrity of the collagen triple helical structure. The typical values for collagen are ~1, while the ratio ~0.75 is typical for gelatin [46]. The ratios of the amide III/1450 cm^−1^ peak intensity range from 1.04–1.10 in the case of the original collagen lyophilizate. Following electrospinning, the values of this ratio decreased sharply to values of 0.68–0.71, i.e., values close to those of gelatin, thus indicating the negative effect of electrospinning on the integrity of the collagen (Figure 8A). The median values evinced a slightly decreasing character over time, i.e., 0.65–0.57 and 0.65–0.60 for the DHT and DHTvac crosslinked collagens, respectively. Following subsequent chemical crosslinking with EDC/NHS, these values stabilized without any significant trends. During chemical crosslinking, the less crosslinked parts of the structure were washed out and, at the same time, the existing structure was preserved with the assistance of newly formed covalent bonds. The study of the ratios of amide III/1450 cm^−1^ following the exposure of the treated materials in distilled water was conducted so as to better approximate to the conditions for the study of the mechanical properties. Electrospun (ES) collagen materials dissolve when exposed to water. The preservation of the collagen structure applying chemical crosslinking also evinced a positive effect after 24 h of exposure in distilled water, at which time the value of the collagen integrity increased. Although the values before and after swelling were statistically significantly different, the increase in the value was moderate (to a max. value 0.74), which indicates that the less crosslinked parts of the collagen structure can still be washed out even during crosslinking. A different situation occurred in the case of physical crosslinking only, concerning which, the values of the ratios of the amide III/1450 cm^−1^ peak intensity increased significantly after 24 h of exposure in distilled water with statistically significant differences for all the DHT application times. These differences were more striking in the case of the application of DHT in the vacuum environment, which indicates that more of the material was less crosslinked and a lower amount of high-integrity collagen remained following exposure to distilled water, which correlates with the “remaining mass” in Figure 4.

The ratios of amides A/I before and after exposure in distilled water for 24 h were determined so as to obtain a better understanding of the various processes that take place within this system. The stretching vibrations of the NH_2_ bonds in the amino acids and the OH bonds in the free and interstitial water are reflected in amide A. The free –NH_2_ groups change to –NH- groups during the crosslinking reaction; the water that is bound to the collagen is lost [47]. Consequently, the integral absorbance of amide A decreases. In contrast, the formation of a new isopeptide covalent bond results in the increasing absorbance of the amide I band. The amide A/amide I area ratio can be used as a factor for the evaluation of collagen crosslinking [48]. A higher A/I ratio indicates that a low proportion of the collagen has crosslinked. Likewise, this ratio reflects the hydration of the given material or the content of other components that contain OH bonds, i.e., in our case, the PEO in the DHT.

The evaluation of the degree of crosslinking using the absolute values of the A/I ratio given the facts described above is often complicated; nevertheless, it is interesting to evaluate the relative A/I ratio before and after exposure in distilled water (Figure 8B). In the cases of DHT, DHTvac and DHT+EDC, a statistically significant increase in the A/I ratio was evident following exposure in distilled water for 24 h, i.e., more water remained (even following lyophilization) bound within the collagen materials than before exposure; thus, the crosslinking process was not perfect. Conversely, in the case of DHTvac+EDC, a statistically significant decrease was observed, i.e., this collagen material exhibited significantly less bound water following lyophilization; thus, it was the most crosslinked material.

## 4. Discussion

The study investigated the impact of atmospheric pressure and vacuum conditions on dehydrothermal crosslinking, particularly in the context of subsequent chemical crosslinking with EDC/NHS, in nanostructured collagen layers fabricated via electrospinning. These nanostructures have huge potential in terms of their use in tissue engineering applications, particularly in the form of scaffolding and surface layers for implants aimed at enhancing osteointegration and cellular colonization [13,49]. However, the electrospinning process acts to disrupt the crosslinks within the collagen, which compromises the mechanical properties [50]. Therefore, it is imperative that effective crosslinking methods are applied that serve to restore the collagen to its native state and ensure its suitability for use in tissue engineering applications.

In order to confirm the effectiveness of the crosslinking process, materials were prepared with differing exposure times, i.e., 3, 6, 12, 24, 48 and 72 h via DHT in both the vacuum and normal atmospheres. In addition, a material that combined DHT with chemical crosslinking using EDC/NHS was prepared for all the exposure times. The controls comprised the source collagen material, a basic electrospun material that was not exposed to crosslinking and an electrospun material that was chemically crosslinked using EDC/NHS [13,51]. Mechanical testing, the material shrinkage test, the swelling and degradation tests, infrared spectroscopy and the SEM analysis of the morphology were applied to describe the degree of crosslinking. Prior to the mechanical testing, the samples were hydrated in a PBS solution for 24 h so as to allow for the observation of whether the mechanical response of the material approached that required for practical use. The material crosslinked via DHT in the vacuum atmosphere attained Young’s modulus values of 0.195 MPa at 6 h to 0.350 MPa after 72 h and ultimate tensile strength values of 0.191 MPa at 6 h to 0.779 MPa after 72 h. The values obtained for the material crosslinked in the air atmosphere were 0.168 MPa at 3 h to 0.313 MPa after 72 h (Young’s modulus) and 0.159 MPa at 3 h to 0.269 MPa after 72 h (ultimate tensile strength). Concerning the materials that were subsequently chemically crosslinked via EDC/NHS, the Young’s modulus values were 1.348–1.798 MPa and 1.224–0.835 MPa, and the ultimate tensile strength values were 0.877–1.677 MPa and 0.330–0.302 at 3–72 h for the vacuum and air environments, respectively. The highest Young’s modulus value attained for the air environment was 1.356 MPa and the highest ultimate tensile strength value was 0.682 MPa after 12 h and 24 h, respectively.

The stress–strain responses were modeled by means of the Fung–Demiray exponential model (3), which provided a good description of the nonlinear behavior of the hydrated collagen nanostructured materials; none of the *R*^2^ were lower than 0.975 with the exception of the DHTvac after 48 h of exposure (*R*^2^ = 0.940). Thus, it can be concluded that the Fung–Demiray model is suitable for the description of the mechanical response of hydrated collagen materials.

The degradation and swelling tests were performed in deionized water for 24 h. All the physical and chemical collagen crosslinking methods were observed to bolster resistance to degradation. The degree of resistance to degradation for the DHT samples increased with the exposure time, while the chemically crosslinked materials evinced almost complete resistance to degradation, retaining almost 96% of the constituent weight. Moreover, the swelling diminished with increasing exposure time with respect to the DHT method under both vacuum and air conditions, thus indicating an escalating degree of crosslinking. Both the DHT and DHTvac methods, when crosslinked in conjunction with EDC/NHS, evinced similar relatively low degrees of swelling.

Polymers show a certain degree of shrinkage during crosslinking and polymerization [52,53]. This shrinkage may cause problems in their applications. It can be seen from the shrinkage test that following the exposure of the DHT to air, shrinkage occurred that was comparable to that resulting from the chemical EDC/NHS crosslinking. After subsequent crosslinking using EDC/NHS, the material returned to its original size; however, it should be stated that the most extensive relative shrinkage that occurred during this process was around 14%. In comparison, the DHT in the vacuum environment did not exhibit a significant change in terms of the area and, following subsequent crosslinking via EDC/NHS, shrinkage was observed of around 8%, which indicated the further crosslinking of the material. In contrast, an increase was observed in the surface area for the DHT in the air environment. This increase in the surface area, taken together with the results of the swelling test, which showed that this material swells less than the DHT under vacuum conditions, indicated that a lower degree of crosslinking had already occurred during crosslinking using EDC/NHS. This lower rate of crosslinking would usually be reflected in a lower rate of shrinkage; however, in this case, the effect was overcome by the swelling of the material, and thus, overall, it evinced an increase in the surface area. This conclusion was further supported by the results of mechanical tests, where, in the case of the DHT in the vacuum environment, following subsequent crosslinking with EDC/NHS, a more significant increase in the Young’s modulus was evident, i.e., 514% than for the DHT in the air environment, i.e., 267%. Similarly, the ultimate tensile strength of the DHT in the vacuum environment increased by 214% compared to 112% in the air environment.

When comparing the various exposure intervals of DHT and DHTvac, it is evident from Figure 1 and Figure 2 that, concerning the mechanical properties, an increase was evident in terms of the Young’s modulus with the exposure time. Interestingly, the presence of air did not significantly affect the value of the Young’s modulus, i.e., the two procedures evinced a consistent gradual increase, as reported in [54]. In contrast, concerning the ultimate strength, the DHTvac exhibited lower values, which were particularly evident at shorter exposure times. Notably, no functional DHTvac samples were available for measurement at the exposure time of 3 h. However, with prolonged exposure times, the samples crosslinked in the air environment attained their maximum ultimate strength values, following which, the increase stagnated. In contrast, the ultimate tensile strength of the vacuum crosslinked samples continued to increase up to the maximum exposure time, finally attaining a three times higher value than the air-access crosslinking. This suggests that free reaction sites become occupied with reaction products in the presence of air that do not contribute to enhancing the strength, whereas they exert no significant impact on the Young’s modulus of elasticity. Assuming that the strength is proportional to the degree of crosslinking, this observation suggests that the unchanged degree of crosslinking can be attributed to access to air.

Meng and colleagues [12,55] demonstrated that collagen subjected to crosslinking using EDC/NHS exhibited notably lower ultimate strength values for hydrated crosslinked collagen, i.e., (0.22 ± 0.02) MPa, than suggested by the findings of this study, i.e., (0.95 ± 0.22) MPa. Conversely, our results are in agreement with those reported by Huang [12,22]. The discrepancies may have arisen due to variations in the concentration of the EDC/NHS crosslinking agent and the specific methodology employed for crosslinking. Previous studies have highlighted that, despite maintaining constant conditions during electrospinning and utilizing the same collagenous solution, differences may be evident in terms of the mechanical properties and structural characteristics of the final materials [51]. In this instance, it was expected that differences in the crosslinking methods exerted the most significant impacts on the outcomes given that identical electrospun materials were employed in all the sample sets. The discrepancies in the absolute values of the measured parameters compared to those in the literature may have stemmed from disparities in the original materials used; nonetheless, the main trends were comparable.

The crosslinking conditions applied to the samples in our study closely resembled those applied by Ming-Che et al. [56] despite the significant differences in terms of the preparation of the base material. Ming-Che et al. reported ultimate strength and Young’s modulus values of (18.0 ± 7.8) MPa and 127.8 ± 51.7, respectively, after 24 h at 140 °C, which significantly exceed the values obtained in our study, i.e., (0.12 ± 0.06) MPa and (0.19 ± 0.02) MPa, respectively. Similarly, their reported values for a crosslinking duration of 72 h were (26.4 ± 5.4) MPa and (280.5 ± 87.9) MPa, respectively, in contrast to our findings of (0.78 ± 0.10) MPa and (0.31 ± 0.03) MPa, respectively. The discrepancies in the ultimate strength and Young’s modulus values were attributed to differences in the structure of the material. Ming-Che et al. utilized thin, 0.28 mm diameter homogeneous extruded fibers, whereas our study employed nanostructured fibrous materials produced via electrospinning, with a diameter of approximately 200 nm. Although the variance in the diameter may have contributed to the divergent results, both investigations revealed a discernible upward trend attributable to dehydrothermal crosslinking.

Whereas the electrospun non-crosslinked control sample completely degraded, the assessment of degradation presented in Figure 4 shows that throughout the 24 h period of immersion in distilled water, even minimal exposure to DHT or DHTvac resulted in considerable resistance to degradation. According to the test conclusions, more than 25% of the original weight remained for DHTvac, while almost 50% remained for DHT. In addition, the swelling test suggested a superior degree of crosslinking, as evidenced by the DHT exhibiting lower swelling in the presence of air [55]. This phenomenon may stem from the occupation of binding sites during reactions that occur in the presence of air, which, subsequently, impedes the binding of water, thus resulting in reduced swelling rates. The FTIR analysis supported these findings by indicating that DHTvac possessed a greater number of available binding sites and exhibited a lower degree of physical crosslinking than the DHT. Taken together, the various analyses underscore the significant influence of air on the dynamics of the DHT process.

The degree of swelling reported by Chen et al. [54], i.e., values of approximately 200% for the material subjected to DHT at 150 °C for 24 h, contrasts significantly with the findings presented in our study, which recorded values of 3799 ± 493%. This disparity can most likely be attributed to the structural differences between the samples. The nanostructured material used in our investigation had a considerably larger surface area than the homogeneous film studied by Chen et al., thereby facilitating the more effective absorption and retention of water within the inter-fibers. Moreover, in line with the observations made by Chen et al., our research also demonstrated a decrease in the swelling ratio with prolonged exposure to DHT, a trend that correlated with the degree of crosslinking.

**Figure 9:**
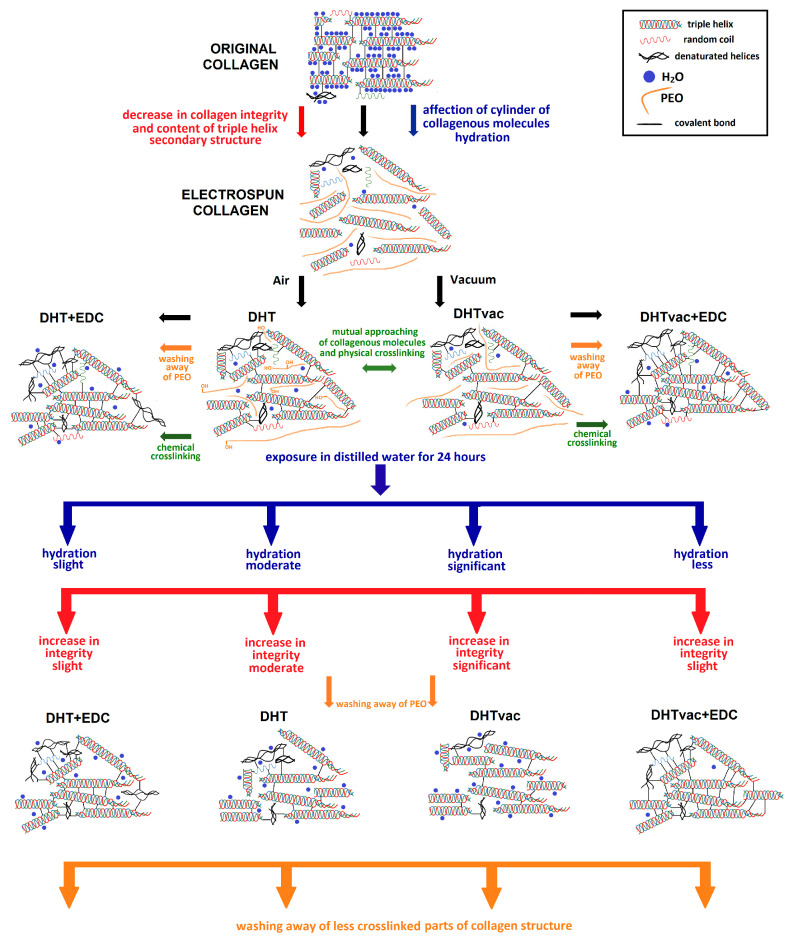
General summary of the reactions within the studied materials; duration of the crosslinking processes = 72 h.

A general summary of the events that took place within the studied materials, proposed on the basis of the results of the structural analysis, is provided in Figure 9. The original collagen was crosslinked by immature and matured covalent bonds [57]. Its integrity at the tertiary structure level was at the level of collagen (1.07—see Figure 8A) with a high proportion of the triple helical component (50%—see Figure S2). The A/I ratio for the original collagen (OR) was around 2.75 (Figure 8B). The content of water in the collagen matrix was influenced by the environmental humidity and by the setting of the equilibrium. Each triple helix was surrounded by a cylinder of hydration (interstitial water) [58] that was responsible for the stabilization of the collagen triple helix via the formation of hydrogen bonds between the helices [59]. The interaction of water and the collagen exerted a significant impact on the water removal dynamics and the final content of the residual moisture in the lyophilizates [60]. Following the electrospinning of the collagen with PEO, this value decreased to 1.83 (Figure 8B). PEO was used in the electrospinning process; as a water-soluble polymer with good biocompatibility and low toxicity, it acts to reduce the surface tension of the spinning liquid, facilitates the splitting of the fibers, and renders the diameter distribution of the fibers more uniform. It can be added to assist in the electrospinning process by increasing the polymer chain entanglements and can subsequently be extracted from the nanofibers via incubation in water so that just collagen remains. Electrospinning influenced the cylinder of hydration of the triple helices together with the slight denaturation of the triple helices (42%) at the level of secondary structure (Figure S2), accompanied by a decrease in the integrity value (0.68) at the quaternary level to that of gelatin (Figure 8A). 

Covalent bonds were formed following the application of physical crosslinking (DHT and DHTvac) due to dehydration. The level of the denaturation of the triple helices remained at a similar level as that following electrospinning (40–42%—see Figure S2) with a slight decrease in the integrity at the quaternary level (∼0.6—see Figure 8A). During the subsequent application of chemical crosslinking (DHT+EDC and DHTvac+EDC), the PEO was washed out (Figure S1). A slight increase in the integrity (∼0.6) was evident at the quaternary level (Figure 8A) with a further increase in the denaturation of the triple helical structure (33–37%) at the secondary level (Figure S2).

Following exposure in distilled water, the PEO was washed out of the DHT and DHTvac materials (Figure S3). Although increases in the collagen integrity values were visible for all the materials (Figure 8A), the increase was most striking concerning the DHTvac physical crosslinking process; the value of 0.87 was close to that of collagen. The increase in the integrity was related to the washing out of the less crosslinked collagen parts. The determination of the degree of crosslinking and hydration using absolute values of the A/I ratio can be complicated. However, according to both the behavior and the changes in the relative A/I ratio before and after exposure in distilled water, it can be deduced that the collagen in the DHTvac material was least physically crosslinked. However, after subsequent chemical crosslinking, the DHTvac+EDC collagen was observed to be the most crosslinked, which correlated with the results of the mechanical tests (Figure 2). The collagen after physical crosslinking in the vacuum environment (DHTvac) contained the largest amount of free bonds, as manifested by the highest degree of hydration following exposure in distilled water (Figure 7B). These free positions in the collagen structure were subsequently available for chemical crosslinking, which is more effective than physical crosslinking. The chemically crosslinked materials (DHT+EDC and DHTvac+EDC) did not evince such a significant increase in their integrity values following exposure in distilled water, i.e., the internal structure was primarily fixed by the covalent bonds that formed during chemical crosslinking.

Chemical crosslinking using EDC/NHS induces the formation of a covalent (amidic) bond between the amino group from lysine or hydroxylysine and the carboxylic groups of aspartic and glutamic acid in collagen; it plays the role of activation agent for two collagen molecules only, i.e., without linkers [30]. The dehydrothermal treatment mechanism suggested the formation of amide crosslinks between the amine and carboxyl groups. However, a further possible alternative crosslinking route was presented via the formation of lysino-alanine throughout the formation of the intermediate dehydro-alanine. Since the basic triple-helical structure of the fiber is retained, this formation depends on the juxtaposition of reactive residues between the molecules. Collagen contains 36 lysine residues per 1000 residues. It is, therefore, possible that a number of such residues were in the wrong position, along the helices of the adjacent molecules, to form crosslinks [16].

## 5. Conclusions

Dehydrothermal crosslinking conducted in the presence of both air and vacuum conditions demonstrated an elevated degree of crosslinking, resulted in increased ultimate tensile strength values, enhanced resistance to degradation and a reduced swelling ratio in the aqueous environment. The FTIR analysis revealed an abundance of free binding sites in the DHTvac, thus indicating a lower degree of crosslinking compared to the other conditions. Observations from both the air-exposed and vacuum-based dehydrothermal crosslinking highlighted an increase in the crosslinking rate with prolonged exposure time, as evidenced by the increasing Young’s modulus and ultimate tensile strength values, along with an enhanced resistance to degradation and a decreased swelling rate. The morphological analysis revealed the enhanced preservation of the fibrous structure in the DHT and EDC crosslinked samples compared to the control using EDC/NHS. Moreover, a distinct difference was noted concerning the DHT with exposure to air: the extended exposure times led to a decreasing Young’s modulus and ultimate tensile strength values, unlike the gradual increase observed in the DHT vacuum environment. The FTIR analysis further highlighted the lower degree of crosslinking in the air-exposed samples. This study sheds new light on the impact of air on dehydrothermal crosslinking by suggesting its potential for shorter exposure intervals. 

The data also suggested that the DHT crosslinking mechanism targets distinct binding sites compared to chemical crosslinking methods. Short-term exposure to air for up to 12 h did not notably affect the material properties when contrasted with DHT performed under vacuum conditions. However, prolonged air exposure led to the degradation of the materials and initiated reactions that occupied the available bonding sites, thereby diminishing the efficacy of chemical crosslinking with agents such as EDC/NHS. This highlights the importance of carefully controlling the environmental factors during the crosslinking process so as to optimize the performance of the materials and preserve the bonding potential for the chemical crosslinkers.

Dehydrothermal treatment takes place under static conditions and, from the above, it follows that the mutual position of the individual collagen fibers (steric factors) comprises a key factor in the formation of crosslinks. In contrast, chemical crosslinking takes place in a dynamic system, in which the molecules of the crosslinking agent are mobile and are, thus, better able to reach the reactive parts of the collagen chains and form crosslinks.

Various dehydrothermal crosslinking conditions can be applied to attain differing target material properties depending on the desired application. In the case of stand-alone DHT, it has been demonstrated that access to air accelerates the crosslinking process compared to vacuum conditions, with the attainment of comparable observed values. This is particularly significant from the economic and technical points of view since vacuum equipment is more demanding in terms of both operation and maintenance. In cases where non-toxic crosslinking conditions are required and where it is not necessary to maximize the material properties, DHT with air access appears to be the preferred option.

In cases where it is necessary to enhance both the mechanical parameters of the material and resistance to degradation, and to reduce the swelling rate, the material must be chemically crosslinked. In this case, the DHT method under vacuum conditions provides the more suitable method since it does not lead to a further decrease in the effectiveness of the chemical crosslinking process.

The data provided by this study suggest that DHT can be used to reduce shrinkage during the crosslinking process. It appears that access to air exerts a significant impact on the shrinkage effect. By combining DHT under vacuum conditions with EDC, it is possible to reduce the maximum shrinkage during the entire crosslinking process from 17% in the case of pure EDC/NHS crosslinking to around 8%. Shrinkage plays a significant role in the material fusion mechanism when applied, for example, as a surface layer for implants or in composite structure applications prepared from this material, which helps to reduce the internal mechanical tension in the material and prevent the appearance of cracks and subsequent deterioration in the case of surface layers or structural failure. 

Additional research is required to elucidate the intricacies of crosslinking reactions that occur in air-exposed and vacuum environments. Forming a comprehensive understanding of these mechanisms has the potential to significantly enhance the economy and efficiency of the production of nanostructured collagen materials, thereby facilitating their application in various medical contexts. Via the more detailed study of the underlying processes involved in crosslinking under different atmospheric conditions, it will be possible to streamline the manufacturing process and unlock novel avenues for medical use. This investigation aimed to bridge a number of existing knowledge gaps and pave the way for the determination of more streamlined and effective production methods for nanostructured collagen materials, thus, ultimately, advancing their medical application potential.

## Figures and Tables

**Table 1:** Summary of the studied materials.

Group	Type of Control/Crosslinking Procedure:
OR (control)	Original collagen material
ES (control)	Electrospun, not crosslinked
EDC/NHS (control)	Electrospun, EDC/NHS crosslinked
DHT	DHT in air
DHTvac	DHT under vacuum conditions
DHT+EDC	DHT in air and subsequent EDC/NHS crosslinking
DHTvac+EDC	DHT under vacuum conditions and subsequent EDC/NHS crosslinking

**Table 2:** Expected values of the parameters of the materials with the confidence intervals and the coefficient of determination (*R*^2^). The Young’s modulus (*E*) and ultimate tensile strength (UTS) values are shown with their sample standard deviation.

	Group	*μ* (MPa)	*±CI μ* 95%	*α* (−)	*±CI α* 95%	*R* ^2^	*E* (MPa)	SD	UTS (MPa)	SD
	EDC	0.352	0.001	3.582	0.022	0.998	0.954	0.019	0.859	0.223
DHT	3 h	0.063	0.001	1.575	0.073	0.976	0.168	0.011	0.159	0.062
	6 h	0.088	0.001	2.841	0.043	0.978	0.229	0.016	0.360	0.107
	12 h	0.111	0.000	2.198	0.014	0.989	0.261	0.011	0.285	0.084
	24 h	0.099	0.001	1.733	0.021	0.976	0.239	0.016	0.265	0.117
	48 h	0.117	0.001	1.902	0.024	0.985	0.293	0.014	0.263	0.017
	72 h	0.123	0.001	1.715	0.024	0.978	0.313	0.031	0.269	0.100
DHTvac	6 h	0.075	0.001	4.342	0.079	0.996	0.195	0.006	0.191	0.052
	12 h	0.087	0.000	3.379	0.056	0.975	0.220	0.017	0.157	0.037
	24 h	0.072	0.001	3.717	0.417	0.976	0.194	0.017	0.124	0.057
	48 h	0.083	0.001	1.262	0.318	0.940	0.224	0.040	0.329	0.248
	72 h	0.135	0.001	3.444	0.110	0.966	0.350	0.036	0.779	0.059
DHT+EDC	3 h	0.431	0.004	6.982	0.247	0.989	1.224	0.076	0.330	0.132
	6 h	0.498	0.003	4.083	0.084	0.989	1.353	0.066	0.691	0.221
	12 h	0.489	0.002	4.133	0.044	0.997	1.356	0.029	0.675	0.117
	24 h	0.392	0.002	2.391	0.031	0.989	1.049	0.096	0.682	0.266
	48 h	0.392	0.004	2.777	0.107	0.981	1.077	0.090	0.478	0.163
	72 h	0.300	0.003	3.087	0.154	0.990	0.835	0.046	0.302	0.087
DHTvac+EDC	3 h	0.494	0.006	7.099	0.306	0.979	1.348	0.105	0.877	0.086
	6 h	0.512	0.005	7.076	0.229	0.984	1.421	0.105	0.928	0.212
	12 h	0.503	0.005	5.475	0.157	0.983	1.490	0.194	1.456	0.230
	24 h	0.527	0.003	3.987	0.073	0.990	1.407	0.046	1.568	0.248
	48 h	0.552	0.004	3.934	0.062	0.988	1.519	0.117	1.742	0.388
	72 h	0.703	0.006	2.772	0.088	0.978	1.798	0.093	1.677	0.387

## Data Availability

The original contributions presented in the study are included in the article/Supplementary Materials, further inquiries can be directed to the corresponding author.

## Supplementary Materials

The following supporting information can be downloaded at: https://www.mdpi.com/article/10.3390/polym16172453/s1, Figure S1: Comparisons of the infrared spectra of collagenous materials exposed to all crosslinking processes in both environment in air and vacuum for 72 h before exposure in distilled water and spectra of PEO. Figure S2: Area of 1660 of original (OR), electrospun (ES) collagens compared with samples treated by DHT, DHTvac, DHT+EDC and DHTvac+EDC processes after 72 h. The Kruskal-Wallis + Dunn’s multiple comparisons test (*n* = 10), statistically significant differences are marked “*”. Figure S3: Comparisons of the infrared spectra of collagenous materials exposed to physical crosslinking for 72 h (DHT and DHTvac) before and after exposure in distilled water for 24 h.
