# Supplementary material for: Comparative Study of the Dehydrothermal Crosslinking of Electrospun Collagen Nanofibers: The Effects of Vacuum Conditions and Subsequent Chemical Crosslinking
Source: Polymers (Basel). 2024 Aug 29;16(17):2453. doi: 10.3390/polym16172453 (PMC11398025; doi:10.3390/polym16172453)
Supplement: Supplementary file 1 [file polymers-16-02453-s001.zip › polymers-3151958-supplementary.pdf]

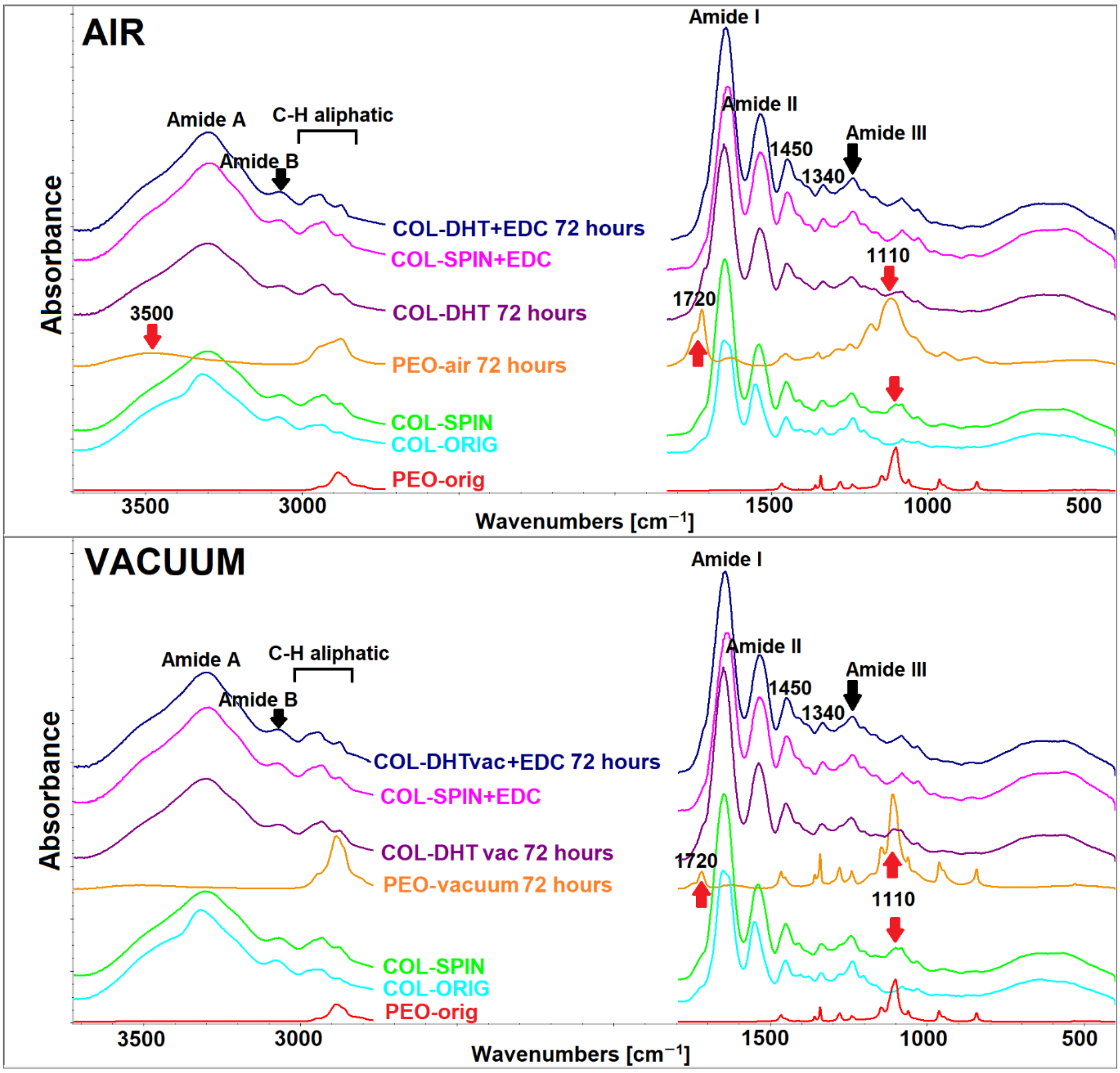

Figure S1. Comparisons of the infrared spectra of collagenous materials exposed to all crosslinking processes in both environment in air and vacuum for 72 hours before exposure in distilled water and spectra of PEO.

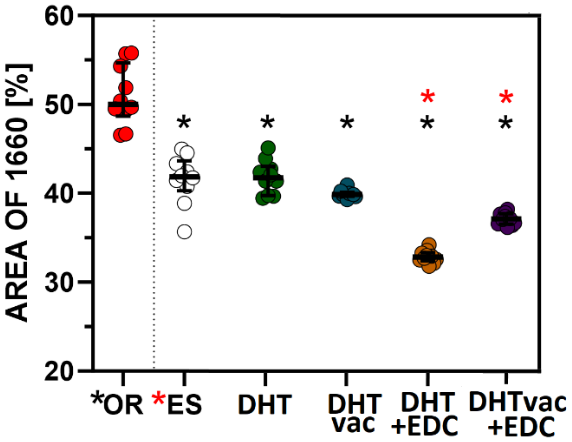

Figure S2. Area of 1660 of original (OR), electrospun (ES) collagens compared with samples treated by DHT, DHTvac, DHT+EDC and DHTvac+EDC processes after 72 hours. The Kruskal-Wallis + Dunn's multiple comparisons test (*n=10*), statistically significant differences are marked "*".

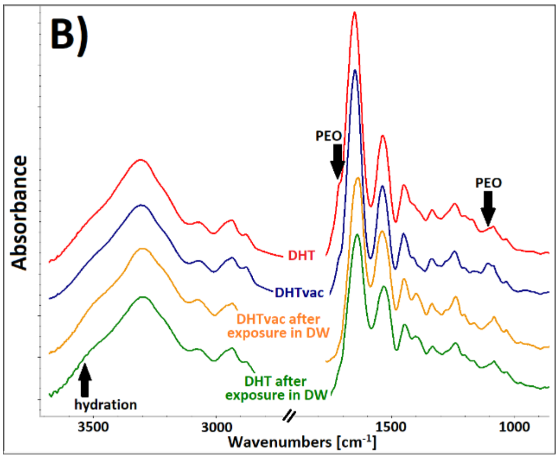

Figure S3 Comparisons of the infrared spectra of collagenous materials exposed to physical crosslinking for 72 hours (DHT and DHTvac) before and after exposure in distilled water for 24 hours.
